# 3D exploration of gene expression in chicken embryos through combined RNA fluorescence in situ hybridization, immunofluorescence, and clearing

**DOI:** 10.1186/s12915-024-01922-0

**Published:** 2024-06-03

**Authors:** Maëlys André, Sarah Dinvaut, Valérie Castellani, Julien Falk

**Affiliations:** MeLiS, CNRS UMR 5284 - INSERM U1314, Université Claude Bernard Lyon 1, 8 avenue Rockefeller, 69008, Lyon, France

**Keywords:** RNA fluorescence in situ hybridization (HCR RNA-FISH), Immunofluorescence (IF), Tissue clearing, ECi, Whole mount chicken embryos, Development, Gene expression, Lightsheet microscopy

## Abstract

**Background:**

Fine characterization of gene expression patterns is crucial to understand many aspects of embryonic development. The chicken embryo is a well-established and valuable animal model for developmental biology. The period spanning from the third to sixth embryonic days (E3 to E6) is critical for many organ developments. Hybridization chain reaction RNA fluorescent in situ hybridization (HCR RNA-FISH) enables multiplex RNA detection in thick samples including embryos of various animal models. However, its use is limited by tissue opacity.

**Results:**

We optimized HCR RNA-FISH protocol to efficiently label RNAs in whole mount chicken embryos from E3.5 to E5.5 and adapted it to ethyl cinnamate (ECi) tissue clearing. We show that light sheet imaging of HCR RNA-FISH after ECi clearing allows RNA expression analysis within embryonic tissues with good sensitivity and spatial resolution. Finally, whole mount immunofluorescence can be performed after HCR RNA-FISH enabling as exemplified to assay complex spatial relationships between axons and their environment or to monitor GFP electroporated neurons.

**Conclusions:**

We could extend the use of HCR RNA-FISH to older chick embryos by optimizing HCR RNA-FISH and combining it with tissue clearing and 3D imaging. The integration of immunostaining makes possible to combine gene expression with classical cell markers, to correlate expressions with morphological differentiation and to depict gene expressions in gain or loss of function contexts. Altogether, this combined procedure further extends the potential of HCR RNA-FISH technique for chicken embryology.

**Supplementary Information:**

The online version contains supplementary material available at 10.1186/s12915-024-01922-0.

## Background

RNA in situ hybridization (RNA-ISH) experiments have provided valuable information to depict gene expression in tissue and whole organisms. This approach has proven essential to discover genes involved in many aspects of embryonic development from tissue and organ patterning to specific cell behaviors.

Many optimizations have been made to improve signal amplification or spatial resolution of RNA-ISH protocol and to enable simultaneous detection of RNAs [1, 2]. Hybridization chain reaction (HCR) RNA-ISH technique allows multiplexed detection of RNAs with excellent signal amplification and high spatial resolution [1, 3, 4]. Third-generation RNA fluorescence in situ hybridization (HCR RNA-FISH) using split initiator probes further increased RNA-ISH specificity by reducing background and false positive [5]. As it provides subcellular resolution and allows multiplexing, HCR RNA-FISH is a powerful approach to study in situ the cellular diversity and novel aspects of gene regulation uncovered by the development of transcriptome analyses at cellular resolution. In the chicken embryo that is a classical model for developmental biology studies, HCR RNA-FISH was for example recently used in combination with single-cell RNA sequencing to describe the progressive specification of neural crest cells or the developmental steps leading to hypothalamus regionalization [6, 7].

In addition to sections, HCR RNA-FISH is also suited for thick samples like whole mount organs and embryos [5–13]. HCR RNA-FISH has been successfully performed on chicken embryos between stage HH4 to HH18 (E0.5 to E3) [5, 7, 8, 12, 14–17]. At later stages, it was only reported for dissected organs and sections [6, 12, 18]. Large and detailed 3D information is important to understand complex cellular interactions during organogenesis or to fully appreciate the topography of systems spanning the entire body like neuronal projections or vasculature. As most embryos are non-transparent, tissue clearing is usually required to get workable 3D information from whole mount embryo images [19–24]. Although chicken embryos are initially transparent, the tissues rapidly gain opacity making the imaging of cells inside the embryo difficult after the second day of development.

**Fig. 1:**
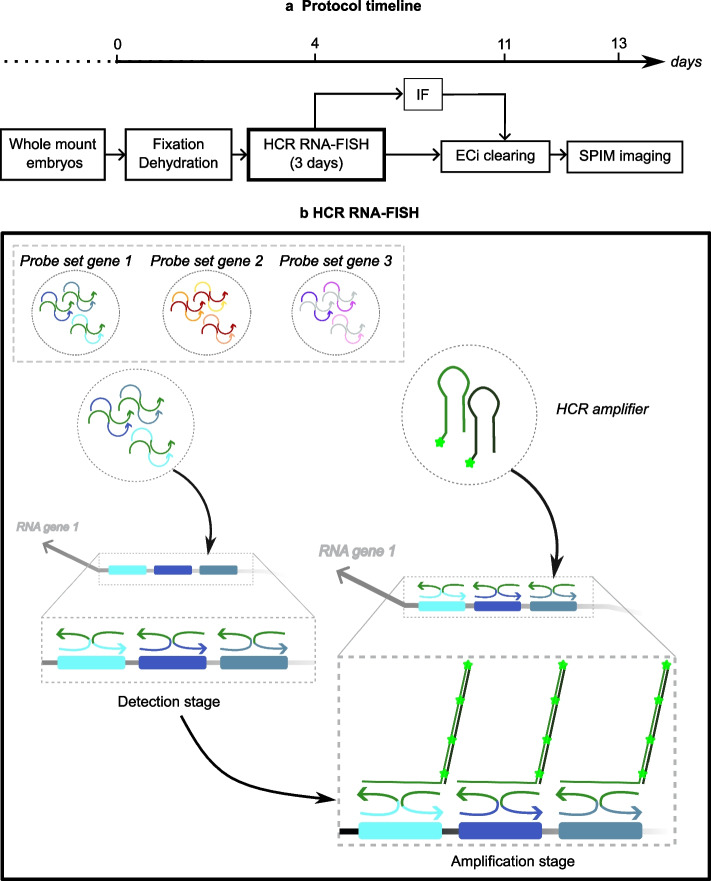
Schematic of the work flow for combining HCR RNA-FISH, immunostaining (IF), and ECi clearing. **a** Timeline of the protocol. The different steps are indicated in boxes and days numbers are shown on the top arrow. **b** Scheme of the HCR RNA-FISH steps adapted from *Molecular Instrument©.* It shows the split-initiator probe pairs used for the detection step (initiator in green, oligos binding to the target mRNA in blue) and the fluorochrome-coupled HCR amplifier that bind to a specific initiator. Stars represent fluorochromes. Different probe sets with different initiators can be used to detect simultaneously different mRNAs with different fluorochromes

In the present paper, we adapted the HCR RNA-FISH protocol for the chicken embryo model from stage HH22 (E3.5) to stage HH27 (E5.5). Next, to get comprehensive gene expressions in whole mount embryos at late stage of organogenesis, we performed ethyl cinnamate (ECi) clearing [25] after multiplexed HCR RNA-FISH. Using different markers, we demonstrate that coupling HCR RNA-FISH with ECi tissue clearing and light sheet microscopy allows the exploration of gene expression with subcellular resolution in whole mount embryos. Finally, we combined HCR RNA-FISH with immunofluorescence on whole mount embryos together with embryo clearing and light sheet imaging (Fig. 1). We show that this combination of techniques can be useful to study spatial relationships between axon and specific cell populations and to monitor gene expression of in ovo electroporated cells.

## Results

### HCR RNA-FISH combined with clearing works on E3.5 chick embryos

As many important neurodevelopmental steps occurs between E3.5 and E5.5, we wondered whether HCR RNA-FISH would work at these stages. The HCR RNA-FISH protocol on chicken embryos proposed by Molecular Instrument© was established for embryos at 1.5 days of development (HH10) [5, 8]. As this protocol was also used on HH18 (E3) embryos [12], we tested this protocol on HH22 (E3.5) embryos. To assay the efficiency of the HCR RNA-FISH, we used probes that target genes having well documented expression patterns. Probe sets were designed by Molecular Instruments and consist in 19 to 20 pairs of oligos recognizing specifically their target mRNA (Fig. 1b). Oligos from different probe set can be coupled to different initiators enabling amplification with specific fluorochromes (Fig. 1b). Thus 2 to 3 probe sets targeting different mRNAs can be hybridized at once on the same embryo.

Multiplexed detection of SRY-box transcription factor 10 (*SOX10*) with ISL LIM homeobox 1 (*ISL1*) and slit guidance ligand 2 (*SLIT2*) or paired box 6 (*PAX6*) with achaete-scute family BHLH transcription factor 1 (*ASCL1*) showed that probe sets gave different labeling (Additional File 1: Fig. S1a, b). Inspection under a stereo-microscope show that signals were found in the expected regions. *SOX10* expression was found in stripes along the dorsal part of the embryo that match with the pattern of migrating neural crest cells, which express *SOX10* [26]. It was also detected in the otic vesicle and in the cranial ganglia that were both reported to express *SOX10* at this stage (Additional File 1: Fig. S1a) [26]. *SLIT2* exhibited a high expression in the ventral spinal cord and hindbrain which is consistent with its known expression at the floorplate and in ventral motoneurons (Additional File 1: Fig. S1a) [27–29]. As expected, it was also detected in the eye and other regions of the embryos [27]. *ISL1* signal was found in ovoid structures along the back of the embryo that correspond to the dorsal root ganglia, which are expressing high level of *ISL1* (Additional File 1: Fig. S1a) [30, 31]. It was also detected in cranial ganglia and in a large ventral spot. This is consistent with *ISL1* in the cranial ganglia and in the developing gut [32, 33]. *PAX6* probes gave a strong signal in the eye that reproduces reported expression in the retina (Additional File 1: Fig. S1b) [34]. *ASCL1* probe labeled a small domain extending from the neck to the tail, in the back of the embryo, which matches with the spinal cord tissue (Additional File 1: Fig. S1b). This labeling is consistent with *ASCL1* expression in a subset of spinal cord progenitors [35, 36]. In conclusion, the different probes appear to give specific signal, which is consistent with the fact that split probes prevent unspecific labeling [5]. In addition, this shows that the published protocol is applicable to whole mount chicken embryo at HH22 enabling the detection of multiple RNAs at the same time.

**Fig. 2:**
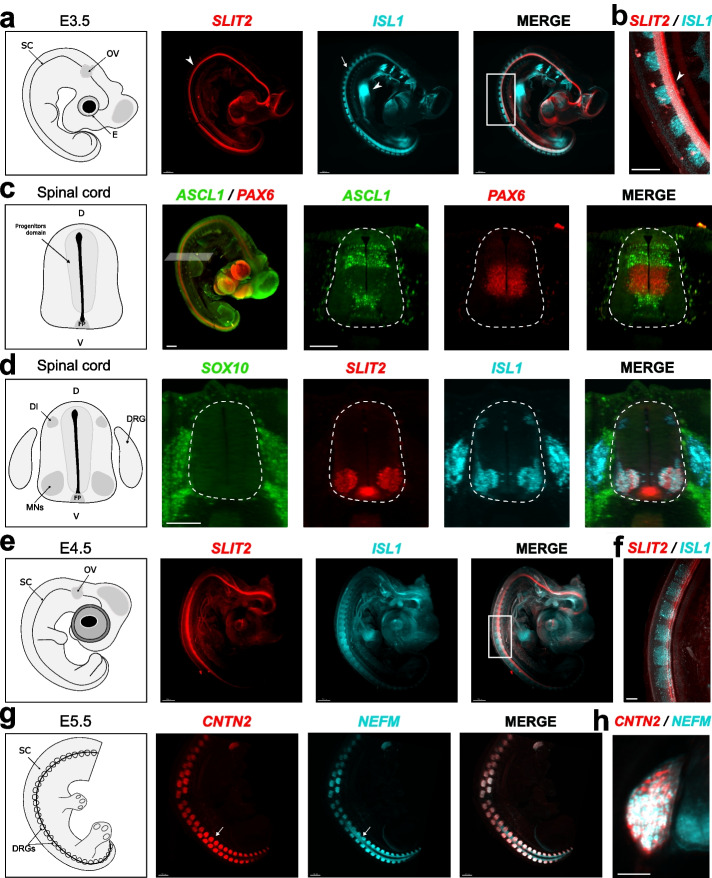
HCR RNA-FISH combined with clearing allows gene expression analysis on E3.5 to E5.5 chicken embryos. **a** RNA expression of *SLIT2* and *ISL1* in E3.5 embryo. *SLIT2* (in red) is detected in spinal cord (SC) (arrowhead) and *ISL1* (in blue) in dorsal root ganglia (DRG) (arrow), pharyngeal arches, otic vesicle (OV), and tissues associated to developing gut (arrowhead). **b** Magnification of merged *SLIT2* and *ISL1* signals shown colocalization in motoneurons (MNs) (arrowhead). **c**, **d** Imaging of cleared whole mount embryos enables examination of gene expression at cellular resolution within tissues. **c** Left panel, 3D images of E3.5 wholemount embryo with the plan of the optical slice presented on the right panels. Virtual coronal section of the spinal cord (dashed outline) shows the complementary expression of *PAX6* and *ASCL1* in the progenitor domain. **d** Virtual section of multiplex HCR RNA-FISH allows to visualize co-expression of *SOX10* and *ISL1* in the DRGs as well as *SLIT2* and *ISL1* in MNs in E3.5 embryos. Specific expressions of *ISL1* in dorsal interneurons (DI) and *SLIT2* in the floor plate are detected, respectively. **e**, **f** Double HCR RNA-FISH on E4.5 embryo illustrating *ISL1* expression in DRGs and its co-expression with *SLIT2* in ventral spinal cord. **g** Co-expression of *NEFM* and *CNTN2* in DRGs (arrows) in E5.5 embryos without head. **h** Coronal optical section of the embryo shown in **g**, illustrating *CNTN2* and *NEFM* expression in the DRG and *NEFM* expression in MNs. Scale bars: 500 μm (**a**, **f**). 200 μm (**b**) 100 μm (**c**, **d**, **g**), 700 μm (**e**)

Nevertheless, due to opacity of the embryo, observations under binocular microscope were limited and did not allow accurate resolution of gene expression in the embryo depth (Additional File 1: Fig. S1a and b). In order to obtain a 3D cellular resolution at the whole organism level, the embryos were imaged by light sheet microscopy after clearing. We used ethyl cinnamate (ECi) clearing procedure [25], which we found to be more efficient on whole mount chicken embryos between HH22 (E3.5) and HH27 (E5.5) than fructose-based See Deep Brain (SeeDB)/FRUIT [37] and has the advantage to involve less toxic reagents than immunolabeling-enabled three-dimensional imaging of solvent-cleared organs (iDISCO) protocols [38]. Although we initially used methanol, the dehydration step before clarification can be done in ethanol. In order to preserve the HCR RNA-FISH signal, samples needed to be post-fixed for 20 min with 4% paraformaldehyde (PFA) before the clearing. When performed on embryos probed with HCR RNA-FISH for *SLIT2, ISL1* and *SOX10* genes, high signal-to-noise ratio was obtained with all probes, and we observed an improved signal compared to non-cleared embryos (Fig. 2a and b, Additional File 1: Fig. S1a). In addition, this technique allowed to observe signals in the depth of embryo. Indeed, 3D volume can be re-sliced and observed after reconstruction using Imaris© software. It is possible to section the same sample in several orientations. For example, virtual coronal sections from a *PAX6/ASCL1* transparized embryo allow us to observe the complementary expression patterns of these genes in different progenitor domains of the spinal cord (Fig. 2c), as expected from previous reports [35, 39]. We made virtual coronal sections of the spinal cord from the 3D reconstruction of the *SLIT2/ISL1/SOX10* embryo indicating the specific expression of these genes in specific domains of the spinal cord and the dorsal root ganglion (DRG) (Fig. 2d). For example, we could detect *ISL1* expression in both ventral (motoneurons) and dorsal (interneurons) neurons (Fig. 2d) that was hidden in whole mount images by its expression in the (DRG) (Fig. 2a and b) [30, 31].

Altogether, we show that HCR RNA-FISH can be coupled to ECi clearing to study gene expressions in HH22 embryos.

### HCR RNA-FISH protocol can be optimized for E4.5 and E5.5 chicken embryos

We found that using the same probes for HCR RNA-FISH on older E4.5 and E5.5 embryos was unsuccessful. The detected signals did not faithfully mirror the expected patterns. For example, *ISL1* expression was low in DRGs and not detected in the dorsal interneurons (GEISHA ISL1.UApcr) [30, 31] (Additional File 1: Fig. S1d). Thus, further optimizations were required to adapt HCR RNA-FISH on older embryos. Therefore, we tested the outcome of different modifications achieved at several key steps of the procedure. They are summarized in Table 1 and will be briefly described below.

**Table 1:** Summary of the optimizations performed on the illustrated probes. The table lists the key steps of the HCR protocol on chicken embryos. Column 2 presents the conditions published for HH10 embryos (E1.5), and column 3 presents the conditions used for E3.5 embryos. Column 4 shows the tests made to find the optimal conditions for E4.5 and E5.5 embryos shown in the last two columns

Steps protocol	HH10	E3.5	*Modifications tests of keys steps*	Validation	
				**E4.5**	**E5.5**
Maximum embryo number per tube	4	3	*2*	2	2
PFA fixation time	1 h at RT	1 h at RT	*Overnight (ON) à 4 °C*	1 h RT	1 h RT
Proteinase K time (min)	2	0	*0–5–15*	0	0
Probes concentration (pmol)	2	2	*2–4*	2–4	4
Hybridization buffer volume (μL)	500	500	*500–1000*	500	500
Probes incubation time	12–16 h	ON	*ON–24*	ON	ON
Dissection	X	X	*Head*	X	Yes

First, we found that it is particularly important during the dissection to eliminate the blood as much as possible to avoid red blood cells, which are not efficiently decolorized (Additional File 1: Fig. S1c) [40]. As they remain opaque, their presence is known to absorb light and generate shadows along the illumination path [40, 41]. They can also increase autofluorescence. Thus, we usually removed the heart. In addition, we recommend to dissect the liver, which is also blood-rich. Second, we observed that cutting the head could improve HCR RNA-FISH. Although head removal benefit was limited for E4.5 embryos (Fig. 2e and f), it was critical to obtain clear and consistent labeling at E5.5 (Fig. 2g and h). Indeed, *ISL1* specific signal in the spinal cord was only detected after head removal at E5.5 (Additional File 1: Fig. S1d).

After dissection, according to the HH10 chicken protocol, the embryos should be fixed in PFA for 1 h at room temperature. However, for E9 mouse embryos (chicken eq. E3.5), fixation is advised overnight at 4 °C [8], and similar fixation has been used on chicken embryos [12]. Thus, we tested whether overnight fixation could improve HCR RNA-FISH on E4.5 and E5.5. No improvement was observed after increasing the fixation time, and, on the contrary, it decreased the clearing efficiency.

Sample permeabilization is often crucial for probes penetration within the tissues. Proteinase K (PK) is commonly used in ISH protocols to improve permeabilization [2]. Depending on the protocol, the incubation time of the PK can change. While the original HCR RNA-FISH protocol on HH10 embryos includes a 2-min PK treatment [8], this step was lengthened to 10 min for HH10 and 30 min for HH18 whole mount embryos [12]. Thus, we tested different PK treatment durations. We found that PK treatment does not improve HCR RNA-FISH signal. Similar conclusions were drawn in other animal model [42], suggesting that, for the short probes used by HCR RNA-FISH, PK treatments is dispensable. Thus, in order to limit variability, we omitted this step.

As it is advised to adjust probe concentrations [8], we compared HCR RNA-FISH efficiency when using 1× (2 pmol) or 2 × concentration for different probes. We found that there is a clear improvement for some, but not for all probes. In our experience, increasing probe concentration seems to be important to reveal low level expressions, as for example the *ISL1* expression in the dorsal spinal cord (Additional File 1: Fig. S1d) and for probes with a low number of oligos. In general, it is certainly important to adapt the concentration to the size of the embryo, the level and number of cells expressing the genes of interest, and the characteristics of the probe. With these modifications, we were able to validate the protocol at E5.5 with various probes including neurofilament medium (*NEFM*) and contactin 2 (*CNTN2*) (Fig. 2g and h).

To conclude, we found that both dissection and PFA fixations (before and after HCR RNA-FISH) are crucial to get optimal labeling and light sheet imaging. With this, HCR RNA-FISH gives reliable and consistent signals on whole mount E4.5 to E5.5 chicken embryos. Embryos could be imaged several times with limited photo-bleaching, and good signals were still observed a year after the first observation (Additional File 1: Fig. S1e). This shows that HCR RNA-FISH is really stable over time.

### 3D imaging of HCR RNA-FISH enables detailed and quantitative analysis of gene expression in different tissues

**Fig. 3:**
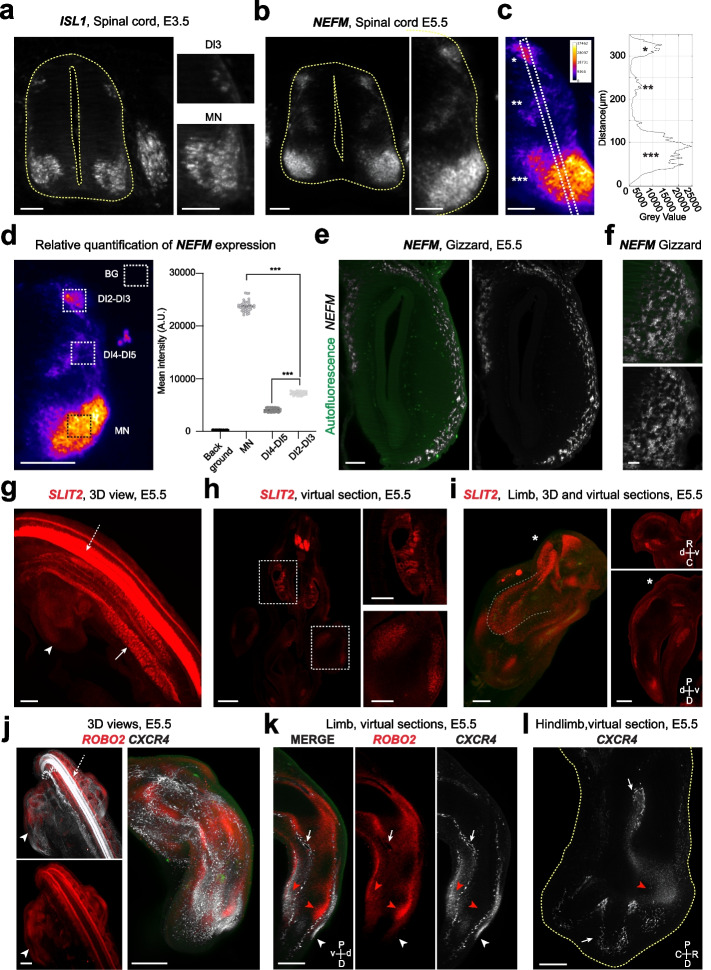
HCR RNA-FISH allows detailed analysis of gene expression in different organs. **a** *ISL1* labeling in the spinal cord of E3.5 embryo. Left panels magnifications of the ventral (MN) and dorsal (DI3) expressing neurons. **b** *NEFM* labeling in the spinal cord of E5.5 embryo. Right panel, magnification of the hemi spinal cord. **c** Fire LUT of *NEFM* labeling illustrating the differences in signal intensity. Right panel, intensity profile along the boxed area shown on the left panel. Asterisks *, **, *** indicate the same regions of interest on the image and the profile. **d** Left panel, example of images quantified showing *NEFM* labeling in Fire LUT and the regions used to quantify background (BG), motoneurons (MN), dorsal interneurons 2–3 (DI2–DI3), and dorsal interneurons 4–5 (DI4–DI5) (dashed boxes). Right panel, scatter plot of the measures made in the different regions. *** indicates *p* = 0.0002 (nonparametric ANOVA) (Additional File 3). Images from **a** to **d** are single Z plans. **e**, **f** *NEFM* HCR RNA-FISH in the gizzard of E5.5 embryos. **e** Virtual transverse section showing that *NEFM* expression is restricted to clusters of cells of the outer layers of the gizzard. The green channel autofluorescence highlights gizzard anatomy and lumen*.* **f** Top views of *NEFM* HCR RNA-FISH at the surface of gizzard. **g** 3D view of *SLIT2* HCR RNA-FISH in a E5.5 embryo. The dashed arrow points to the ventral spinal cord, the plain arrow to the metanephros and the arrowheads to the limb. **h** Virtual section showing the SLIT2 signal in different regions of the embryo. Right panels are magnifications of the boxed regions corresponding to metanephros (top) and limb (bottom) shown in the left image. **i** *SLIT2* labeling in the developing limb. 3D reconstruction of the limb is shown on the left. Dashed line follows the dorsal horseshoe pattern of SLIT2 expression in the medial part of the limb. Virtual cross (top) and longitudinal sections (bottom) are shown on the left. Asterisks indicate shoulder position. **j** 3D views of *ROBO2* (red) and *CXCR4* (white) HCR RNA-FISH signals in embryo (left) and in the limb (right). The dashed arrow points to the ventral spinal cord and the arrowhead to the limb. **k** Virtual longitudinal section of the limb labeled with *ROBO2* (red) and *CXCR4 (white).* Autofluorescence (green) was added to visualize the surface of the limb. **l** Virtual section showing *CXCR4* expression in the hindlimb. Red arrowheads point to muscle masses, arrows to cells potentially associated to developing blood vessels (**k** and **l**) and the white arrowhead to the subectodermal mesenchyme (**k**). Note that *CXCR4* pattern differs in muscles and presumptive developing blood vessels*.* Yellow dashed lines outline the spinal cord (**a** and **b**) and the limb (**l**). Crosses indicate tissue orientation (R, rostral; C, caudal; d, dorsal; v, ventral; D, distal; P, proximal)*.* Scale bars: 50 μm (**a**, **b**, **c**, **f**), 100 μm (**d**, **e**), 200 μm (**i**, **k**, **l**), 300 μm (**g**, **h**, **j**)

HCR RNA-FISH should provide subcellular resolution of mRNA distribution [4, 5]. Thus, we looked in more detail at the signals we could image with light sheet microscopy. In the spinal cord, *ISL1* HCR RNA-FISH resolved individual cells in the motoneuron (MN) pools and in the dorsal spinal cord (DI3 neurons) at E3.5 (Fig. 3a). As they differentiate, neurons progressively express a neuronal-specific intermediate filament called neurofilament medium (*NEFM*) [43]. At E5, both ventral motoneurons and dorsal interneurons should have differentiated [44]. *NEFM* HCR RNA-FISH identified different groups of spinal neurons (Fig. 3b). The size and medio-lateral positions of the ventral (MN) and the different dorsal interneurons were consistent with those expected. *NEFM* signal was clearly cytoplasmic (Fig. 3b). Thus, light sheet microscopy allows fine characterization of gene expression.

Closer examination also showed that *NEFM* labeling intensity differed between neurons subtypes (Fig. 3b). Fire LUT and intensity profile exemplify the high dynamic range achieved with our imaging and confirm the differences (Fig. 3c). Neuronal maturity is expected to vary among different spinal neurons because their differentiation rate and onset differ [44]. Interestingly, single-cell RNA sequencing of human fetal spinal cord showed that neuronal maturity correlates with *NEFM* expression levels [45]. Indeed, the web application provided by the authors shows that *NEFM* expression is low in dorsal interneurons except in the DI3 population and high in MNs [45]. Thus, variation of *NEFM* signal could reveal pertinent differences.

As HCR RNA-FISH signals scale proportionally to target number, relative quantification of mRNA abundance can be done [5, 13]. The voxel resolution of our images is close from the one previously used [5, 13]. We measured in one embryo the signal intensity in different dorsoventral regions of the spinal cord. Measures were made on 2 groups of 21 images covering 30 μm and separated by 120 μm. They showed that background variation was low and that consistent differences could be seen between MN and DI3 and between DI3 and other dorsal interneurons (Fig. 3d). The quantification matched the expected relative variations with the MN exhibiting the highest signal followed by DI3 neurons (Fig. 3d). These data show that relative quantitation is achievable with our method.

Next, we asked whether all regions of the embryos could be imaged with the same resolution. *NEFM* is also expressed by the enteric neurons, which cover the digestive tract. Gizzard is a large, easily identifiable structure where neuronal differentiation occurs early during development [46]. Specific *NEFM* labeling was observed in the superficial layer of the gizzard (Fig. 3e). Top view of the surface of the gizzard further shows that *NEFM* signal could be well resolved in this structure too (Fig. 3f). This supports that detailed analysis of gene expression is achievable in different regions and organs of the embryo.

To investigate this further, we took advantage that genes of interest for spinal cord development, such as *ISL1* or *SLIT2*, are also expressed in non-neuronal tissues. As shown before, we could detect *ISL1* or *SLIT2* expressions outside of the nervous system (Fig. 2a and e). We explored *SLIT2* expression more comprehensively. 3D view revealed its complex pattern of expression and virtual sections enabled detailed analysis of its expression (Fig. 3g and h). As expected [27], *SLIT2* signal was detected in the metanephros (kidney) and the developing limb (Fig. 3g and h). In the limb, *SLIT2* signal distributed in horseshoe pattern in both the dorsal and ventral mesenchymes that was easily identifiable from 3D reconstructions (Fig. 3i). SLIT2 was also expressed in the peripheral mesenchyme in anterior and posterior regions (Fig. 3i). Virtual sections confirmed that *SLIT2* expression domains correspond to ventral and dorsal muscle masses and that *SLIT2* was not expressed in the subectodermal mesenchyme (Fig. 3i). This pattern was highly consistent with previous work describing SLIT2 expression in the developing limb of chicken embryos [27, 47]. C-X-C motif chemokine receptor 4 (*CXCR4*) and roundabout guidance receptor 2 (*ROBO2*), which are expressed in the spinal cord, are also known to be expressed in developing limb of chicken embryos [47, 48]. In agreement, we detected *CXCR4* and *ROBO2* mRNAs in the spinal cord and the developing limb (Fig. 3j). Although their expression differed, virtual longitudinal section of the limb showed that *CXCR4 and ROBO2* are both expressed in some regions of the premuscle masses and of the subectodermal mesenchyme (Fig. 3k). *CXCR4* signal appeared diffused in muscle and subectodermal regions. However, it also labeled individual cells, which aligned in the limb and decorate peri digit domains in the footplate (Fig. 3k–l). These cells could correspond to developing blood vessels, which are known to express *CXCR4 *[48]*.*

Altogether, these data show that light sheet imaging of HCR RNA-FISH provides good spatial resolution and signal dynamics making possible to study gene expression in different embryo regions and organs.

### HCR RNA-FISH can be combined with an immunofluorescence and clearing on chicken embryos

Imaging signals from transcripts that primarily distribute in the cell soma fail to capture information about cell morphology and subcellular compartments. Thus, we attempted to integrate immunostaining into our protocol of HCR coupled with light sheet microscopy.

We focused on the neuron-specific neurofilament medium (NF-M) that labels axons and can serve as a relevant marker to analyze the axonal trajectories. Wholemount immunofluorescence (IF) procedure was adapted for E3.5 to E5.5 chicken embryos from Renier and collaborators protocol [38] (see the “Methods” section). Wholemount IF requires a long incubations time with antibodies and buffers that can affect RNA stability. Thus, HCR RNA-FISH is usually performed first [49, 50]. We found that a 20-min post-fixation before immunostaining is required to preserve the in situ hybridization signal.

**Fig. 4:**
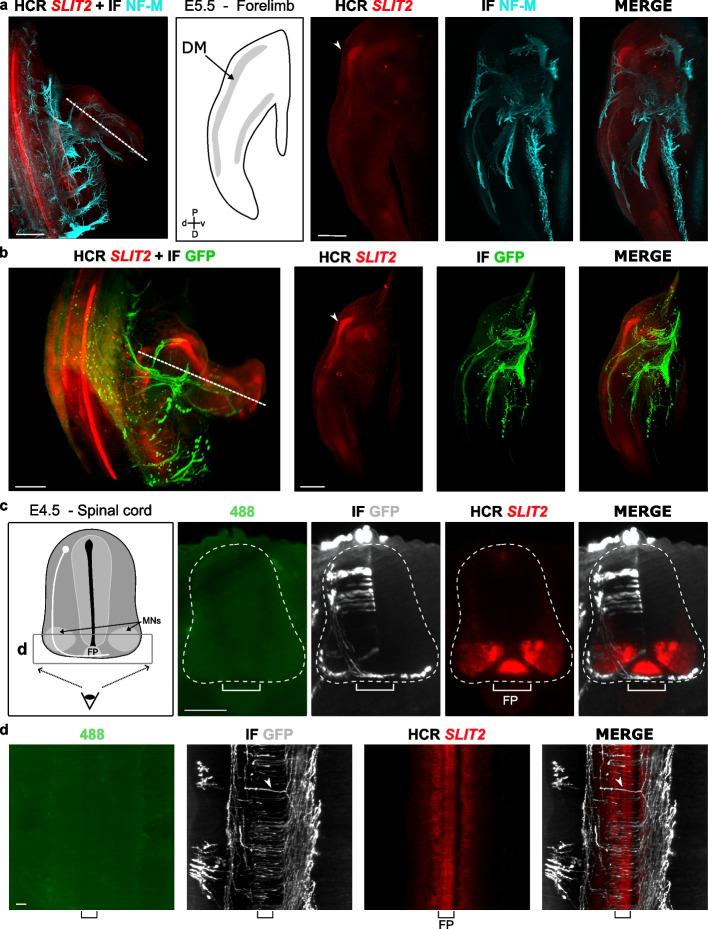
Immunostaining can be done after HCR RNA-FISH on whole mount embryos. **a**, **b** Neurofilament (NF-M) and GFP immunostaining reveals peripheral nerves and respectively transfected axons innervating the limb of SLIT2 HCR RNA-FISH labeled wholemount the E5.5 embryo. Left panels show the 3D images, dashed lines indicate the plan used for the optical section shown in right panels. Schematic drawing of a limb section shows dorsal muscle mass (DM) position and tissue orientation (d, dorsal; v, ventral; D, distal; P, proximal). The relationship between *SLIT2* expression detected by HCR RNA-FISH in the limb DM (arrowhead) and the axons labeled with NF-M (right **a**) or GFP (right **b**) can be analyzed. **c** Virtual coronal sections through the spinal cord (dashed outline) of GFP electroporated embryo with *SLIT2* HCR RNA-FISH and GFP immunolabeling. Schematic drawing of spinal cord shows the dorsal interneuron axons trajectory (white) and the eye indicates the angle used in **d**. **d** Horizontal optical section of ventral spinal cord showing GFP axons crossing the *SLIT2* expressing floor plate (FP). Arrowhead points to an individual axon. Scale bars: 500 μm (**a**, **b**), 300 μm (right panels **a**, **b**), 100 μm (**c**), 30 μm (**d**)

With these adjustments, NF-M staining of axons could be performed on E5.5 embryos with a HCR RNA-FISH for *SLIT2*. *SLIT2* is a repulsive molecule for growing axons and was found to be expressed in limb regions that are not invaded by axons [47]. Peripheral axons distribution could be monitored in 3D with neurofilament immunostaining on cleared embryo after HCR RNA-FISH procedure (Fig. 4a). Thus, we were able to analyze axons position relatively to *SLIT2* expression domains and could show that peripheral axons avoid *SLIT2* regions (Fig. 4a). This confirms the observations made by Vargesson and collaborators [47]. IF procedure did not decrease HCR RNA-FISH labeling durability and IF signal was also stable over time (Additional File 1: Fig. S1f and f’).

This shows that HCR RNA-FISH can be coupled with immunolabeling in chicken embryos, a combination already performed in other models [49–51]. This further increases the potential of this technique to reveal complex spatial relationships and to visualize gene expression and cell morphology at the same time.

Methanol dehydration is known to quench green fluorescent protein (GFP) fluorescence [52]; as a result, GFP signal is progressively lost after HCR RNA-FISH and ECi clearing (Additional File 2: Fig. S2a). Thus, IF could be useful to reveal GFP after HCR RNA-FISH. Electroporation has been widely used to label and genetically manipulate embryonic neurons to study their generation and axon guidance [53–59]. In this context, GFP is often used to monitor cell morphologies or identified transfected cells [53, 60–63]. We performed HCR RNA-FISH on GFP electroporated embryos. The neural tube of HH14 embryos was electroporated with a GFP plasmid. The embryos were collected 2 or 3 days later. *SLIT2* HCR RNA-FISH and GFP immunostaining were performed. Immunolabeling was specific and HCR RNA-FISH signal preserved (Additional File 2: Fig. S2b and c). Embryo examination by light sheet microscopy showed that we can efficiency monitor the morphology of GFP transfected neurons after HCR RNA-FISH. Similarly to NF-M IF, we could observe GFP positive axons navigating along the border of *SLIT2* expressing domain within the forelimb (Fig. 4b). As expected, in the spinal cord, GFP positive axons of the dorsal interneurons were observed to cross the midline below the soma layer of the floor plate that strongly expresses *SLIT2* (Fig. 4c) [64]. The resolution even allowed us to observe the individual axons navigating across the floor plate (Fig. 4d).

Thus, the application of HCR RNA-FISH on electroporated embryos is well suited to analyze gene expression in cell that have complex morphologies and its relationships with their differentiation which can be insightful to study neuron, muscle or endothelial differentiation.

## Discussion

We established a robust HCR RNA-FISH protocol enabling 3D exploration of gene expression in chicken embryos from stage E3.5 to E5.5. This further extends the use of the HCR RNA-FISH protocol [5, 12] and illustrates the potential of the technique to characterize complex gene expression patterns. HCR RNA-FISH optimization and combination to clearing and immunostaining expands the current applications of this technique to novel questions of developmental biology.

We showed that the ECi is a fast and yet efficient clearing method compatible with HCR RNA-FISH. This adds ECi to the other clearing methods including Clearing-enhanced 3D [49], iDISCO [51], or fructose-glycerol [9] that have been used so far with HCR RNA-FISH in other animal models. Each tissue clearing method has its advantages and drawbacks [24]. However, we found that ECi was a good compromise in terms of efficiency, experimental convenience and duration. Consistently with other reports [24], we found that FRUIT was less efficient than ECi and iDISCO. As with many transparization methods, blood cells and eye pigments are not decolorized with ECi. This leads to light abortion and scattering. To limit this, we chose to optimize blood elimination during dissection and to remove the heart. With this, we found that H_2_O_2_ beaching was dispensable. However, decolorization methods might improve tissues clearing if blood cells are present [24, 40, 65]. Reduction of autofluorescence in the green channel is another potential optimization of the present protocol. Interestingly, a photochemical bleaching method was recently combined with HCR RNA-FISH and clearing on developing mouse limbs [66].

HCR RNA-FISH on cleared embryos is fast and efficient way to study gene expression at cellular levels in wholemount embryos. We tested different probes and could reproduce previously published patterns. 3D reconstruction and virtual sections gave complementary information. The possibility to slice the volume in any desired orientations facilitated the analysis and the comparison with previous works. As HCR RNA-FISH signals do not diffuse [4, 5], the signals are clear and well-defined, allowing cells expressing the transcript of interest to be identified and located. Using different probes, we showed that light sheet imaging of HCR RNA-FISH could be useful to explore different tissues and organs. One potential limitation of the technique is due to the light sheet minimal thickness that sets the maximal Z resolution. In contrast to classical confocal microscopy, the Z resolution is fixed. For the Blaze microscope, the light sheet minimal thickness is 3.86 μm. Thus, to resolve fine structures, one has to orient the correctly the embryo to benefit from the XY resolution provided by the ×4 and ×12 objectives. Future development of deconvolution might improve Z resolution [67–69].

HCR RNA-FISH was shown to enable two types of quantitative analyses of the expression levels (single molecule detection and relative quantitation) [5]. Single molecule quantification is not feasible with our method. Indeed, embryo shrinkage during clearing and light sheet imaging resolution (notably in Z) prevent single molecules quantification. In addition, single molecule detection requires short amplification times to separate signals from different molecules [5]. Whether short amplifications would work on E3.5 to E5.5 embryos is not known, but it is likely that it will not generate a uniform marking in the embryo [13].

Nevertheless, the resolution and the dynamic range of light sheet imaging is compatible with relative quantification of gene expression [5, 13]. Overnight amplification should provide sufficient time for the signal to plateau and to ensure homogeneous labeling within the embryo, which is essential for relative quantification [13]. Relative levels of expression are only possible for signals whose intensity is significantly above that of background [5]. Thus, red and far-red channels should be preferred. We do not know the minimal level of expression that can be detected and reliably quantified. However, it is likely as stated by Choi and collaborators that variations of low copy targets will be difficult to assay [5]. To perform relative quantitation, some precautions must be taken during the imaging. First, quantification need to be done on voxels of similar size. Light sheets are usually flat over a small part of the image. Thus, to ensure similar Z resolution, “dynamic focusing” function should be used [70]. Second, light sheet intensity can also decrease when entering into the sample leading to variations in illumination between the two sides. If this is the case, using light sheets from both sides is necessary. Illumination inhomogeneity could be evaluated and corrected.

By measuring *NEFM* signal intensity, we showed that variations in gene expression could be quantified from HCR RNA-FISH images acquired with a light sheet microscope. Although the quantification we provided is relatively crude, it showed that signal-to-noise ratio is good and that labeling intensity is consistent over at least a hundred microns allowing reliable quantification. Intensity variations of *NEFM* labeling were consistent with our knowledge of neuronal differentiation in the spinal cord and recent single cell RNA sequencing data [44, 45]. This illustrates that pertinent information about expression levels could be measured with our method. This suggests that the technique would be suitable to validate single cell RNA sequencing data. It could also be useful to evaluate change of expression after embryo manipulation, providing that changes are large enough. We did not estimate the minimal differences that could be measured with our method, but it is known that HCR RNA-FISH could detect a twofold change in transcripts [13]. Thus, HCR RNA-FISH may be suitable to evaluate knock-down efficiency since it has better spatial and quantitative features compare to classical in situ hybridization that have been used in the past [71].

More generally, we think that combining HCR RNA-FISH, IF, and light sheet imaging of cleared embryos can be useful for functional studies. First, HCR RNA-FISH has proven to be useful for functional studies. HCR RNA-FISH was recently used to assay the impact of BMP morphogen on specific progenitors of the developing hypothalamus in younger chicken embryos [18]. As our method gave good specificity, intensity, and spatial resolution, it should apply equally well to functional studies. Second, the 3D analysis could facilitate phenotype analyses. This is was shown in chicken embryos, for example to, study axon trajectories in the limb and the spinal cord [71, 72]. It also facilitates the detection of phenotypes that would be difficult to identify otherwise [73]. Indeed, 3D reconstructions reveal complex spatial organization that cannot be pictured in 2D [73]. Third, our work showed that the combination HCR RNA-FISH and IF can provide important information for functional studies. We could show that, in the limb, peripheral axons navigate along the borders *SLIT2* expressing regions without entering into them. This confirms the observations of Vargesson and collaborators [47] and would be consistent Slit2 acting as repulsive cues during early stage of limb innervation.

We anticipate that performing IF after HCR RNA-FISH on whole mount embryo can be very useful. IF labeling with well recognized markers can provide important cyto-architectonic context to facilitate the localization of the expression at the organ level [51]. At cellular level, IF counter-labeling allows to assay the expression of genes within specific cells and to correlate gene expression and morphological differentiation. After in ovo electroporation, IF can be used to monitor transfected cells to study their differentiation and morphology more easily in normal but also gain and loss of function contexts. By allowing the detection of protein and RNA expressions in the same cells, IF labeling combined with HCR RNA-FISH might be particularly helpful to study post-translational regulations playing instrumental roles during cell differentiation and axon development [74–77].

## Conclusions

The present combination of techniques provides good sensitivity and sufficient spatial resolution to enable analysis of gene expressions in their 3D contexts. Although light sheet imaging has some limitations and that optimizations can still be made to further increase signal-to-noise ratio, our method gave workable, consistent and stable labeling.

This method is relatively fast as 2 to 3 mRNAs could be detected at the same time and that ECi clearing only takes 24 h. In addition, light sheet microscopy is also time-effective, allowing fast imaging of large objects, which can be virtually sliced afterward, saving tedious sectioning works. HCR RNA-FISH provides very good cost-effectiveness ratio as it is possible to pool several embryos in the same tube. We also found that in most cases the quantity amplifiers can be reduced by 2 times. It also has the advantage of being as economic as a slide-based protocol. Compared to imaging on slices, 3D imaging enables fast exploration of cell distributions and positions as well as better comprehension of their 3D arrangements.

## Methods

### Animals

Embryonated eggs (naked neck strain) were obtained from the Elevage avicole du Grand Buisson (Saint Maurice sur Dargoire, France) and kept at 18 °C until used. They were then incubated at 38.5 °C in a humidified incubator (Sanyo, MIR-154) until they reached the desired developmental stages [78]: HH14 (52 h of incubation) for electroporation, HH22 (E3.5, 3.5 days of incubation), HH24 (E4.5, 4.5 days of incubation), and HH27 (E5.5, 5.5 days of incubation) for other experiments. According to the revised European ethics legislation (2013), experiments did not require specific protocol approval by ethic committee as they were performed within the 6 first days of development.

### HCR RNA-FISH

All probes were designed and purchased from Molecular instruments (Table 2). The protocol was adapted from the Molecular instruments’ protocol for HH10 chick embryo (https://files.molecularinstruments.com/MI-Protocol-RNAFISH-Chicken-Rev10.pdf) [8]. The 3.5 to 5.5 days chicken embryos (*Gallus gallus*) were dissected in Dulbecco phosphate-buffered saline calcium chloride and magnesium chloride free (DPBS, Gibco, 14190250) and collected in plate on ice. The yolk membrane, heart, and liver were removed. Dissected embryos were washed in ice cold DPBS before being fixed in 4% paraformaldehyde for 1 h at room temperature (RT). PFA solution was prepared extemporaneously in PBS (phosphate-buffered saline tablets, 18912–014) using 32% PFA stock solution (Electron Microscopy Sciences, 15714). Embryos were washed twice in PBS-Tween 0.1% (Tween 20X, Electron Microscopy Sciences, 25564) before being transferred in tubes. They were dehydrated in 20 min (E3.5/E4.5) or 30 min (E5.5) methanol (MetOH, Sigma-Aldrich, 322415-1L)–PBS tween (PBST) bath series on ice: 25% MetOH/75% PBST, 50% MetOH/50% PBST, 75% MetOH/25% PBST, 100% MetOH. An additional 1 h incubation in 100% MetOH at RT was done before storing the embryos at – 20 °C for at least one night. Embryos were rehydrated for 20 min (E3.5/E4.5) or 30 min (E5.5) in MetOH/PBST bath series on ice. Embryos were post-fixed in 4% PFA for 20 min at RT. On ice, the embryos were washed for 5 min, twice in PBST, then once in 50% PBST–50% saline-sodium citrate with Tween 20X (SSCT) (Saline-Sodium Citrate, Sigma-Aldrich, S6639 with 0.1% Tween 20X), and finally once in SSCT.

**Table 2:** List and characteristics of the HCR probe sets used. Each probe pair binds to a 52-nt specific sequence on the target

Probes	Accession (NCBI)	Probe set size	Design	Initiator	Amplifier-fluorochrome
*ASCL1*	NM_204412.2	19	Custom	B3	B3-Alexa 488
*CNTN2*	NM_001004395.2	20	Custom	B2	B2-Alexa 546
*CXCR4*	NM_204617.2	20	Probe set catalog	B1	B1-Alexa 647
*ISL1*	NM_205414.2	20	Custom	B1	B1-Alexa 647
*NEFM*	NM_001101730.2	20	Custom	B1	B1-Alexa 647
*PAX6*	NM_205066.2	20	Probe set catalog	B2	B2-Alexa 546
*ROBO2*	XM_040659052.1	20	Custom	B2	B2-Alexa 546
*SLIT2*	NM_001267075.2	20	Custom	B2	B2-Alexa 546
*SOX10*	NM_204792	20	Probe set catalog	B4	B4-Alexa 488

The embryos were pre-hybridized in 500 μL of hybridization buffer for 30 min at 37 °C (HCR™ Buffers, Molecular instruments). The pre-hybridization solution was replaced by the solution with the probes (2 pmol or 4 pmol in 500 μL hybridization buffer) and incubated overnight at 37 °C with shaking (Incu-shaker Mini, Benchmark, H-1001-M-E). After removal of the probe solution, samples were washed 4 times in 1 mL of washing buffer (HCR™ Buffers, Molecular instruments) for 15 min at 37 °C with shaking. As recommended, two 5 min washes in SSCT solution were made at RT. Samples could be stored at 4 °C until the amplification step (up to 72 h). Next, the embryos were incubated in 500 μL of amplification buffer for 5 min at RT (HCR™ Buffers, Molecular instruments). The hairpins h1 and h2 (30 pmol 10 μL for 500 μL of buffer) were heated separately at 95 °C for 90 s, left at RT for 30 min minimum in the dark before being added in 500 μL of amplification buffer. The pre-amplification solution was replaced by the solution containing the amplifiers and incubated overnight in the dark at RT with gentle agitation (Polymax 1040, 543–42205-00). Amplifier solution was removed and embryos were washed in SSCT at RT (2 × 5 min, 2 × 30 min, 1 × 5 min). They were post-fixed in 4% PFA for 20 min and washed twice in PBS. Samples were stored at 4 °C protected from light.

We usually performed HCR RNA-FISH with 2 embryos per 2 ml tube. To avoid contamination, filtered tips were used. A control without probes was performed for all amplifiers used for all the different stages tested to evaluate autofluorescence and unspecific amplifier signal. Sample manipulation was done under chemical hood for the steps that used MetOH, PFA, hybridization, and wash buffers. All solutions were stored at – 20 °C. Probes (10 μl, 5 to 10 tubes) and amplifiers (20 μl, 2 tubes) were aliquoted and defrosted on ice before each use. The solutions were homogenized before use by pipetting.

### Immunofluorescence

The samples were incubated in blocking solution (phosphate-buffered saline 1X (PBS, Gibco, 18912014), glycine 100 mM (ROTH, ref. 3908.2), dimethyl sulfoxide 20% (DMSO, Sigma-Aldrich, D8418-250ML), Triton 0.5% (Sigma-Aldrich, T9284-500ML), and bovine serum albumin 3% (BSA; Sigma-Aldrich, A7906-500ML) at RT during 24 h. Next, the primary antibodies (anti-NF-M; anti-GFP, ref. Table 3) diluted in blocking solution were incubate at RT for 3 days. Embryos were washed 5 times in PBS1X containing 2% DMSO and 0.5% Triton (wash solution) at RT over a day (8 h). The samples were incubated with secondary antibodies (anti-mouse; anti-rabbit, ref. Table 3) in blocking solution overnight at RT. Antibody solution was removed, and samples were rinsed 5 times in the wash solution (1 h at RT). All incubations were done in the dark on roller mixer (SRT6D, Stuart) at low rotation speed (34 rpm). At the end, embryos were post-fixed in 4% PFA for 10 min and washed twice in PBS.

**Table 3:** Reference and dilution of the antibodies used

Protein/antigen	Clone	Species (+ dye)	Dilution	RRIDs	References
Neurofilament 160 kDa anti-NF-M	RMO270	Mouse monoclonal IgG	1:500	AB_2532998	Life techno, 130,700
Anti-GFP		Rabbit polyclonal IgG	1:500	AB_221569	Invitrogen, A11122
Anti-mouse IgG		Donkey (Alexa 555)	1:500	AB_2536180	Life techno, A31570
Anti-rabbit IgG		Donkey (FP 647)	1:500		Interchim, FP-SC5110

### Whole mount clearing

After the HCR RNA-FISH or immunostaining step, the samples were dehydrated using a 30-min series of MetOH/H2O washes at RT: 20%, 40%, 60%, 80%, 100%, and last one in 100% methanol overnight. The next day, they were incubated 3 h in MetOH 100%. MetOH was removed, and embryos were incubated in ethyl cinnamate (ECi, Sigma-Aldrich, 112372-100G) for 1 h at RT before changing the bath with a clean ECi. Samples can be imaged after a few hours or the next day. Ethanol clearing alternative is possible with dehydration in EtOH/H2O baths (30 min for each): 100% H_2_O, 50% EtOH, 75% EtOH, 100% EtOH × 2, 100% EtOH (3 h). Embryos were washed in ECi and incubated overnight in ECi at RT under agitation (Polymax 1040, 543–42205-00) before light sheet microscopy. Samples can be stored at RT in the dark during several months.

### Light sheet imaging

Cleared embryos were glued on holders and immersed in ethyl cinnamate bath. Samples were imaged on a light sheet microscope, UltraMicroscope Blaze (Miltenyi Biotec) equipped with × 1.1 (NA = 0.1), × 4 (NA = 0.35), × 12 (NA = 0.53) objectives. Additional lenses could be used to adjust magnification (× 0.6, × 1.6, and × 2). Images were acquired on 5.5 Megapixel sCMOS camera. The XY resolution was 4.8 μm, 1.62 μm, and 0.54 μm for the × 1.1, × 4, and × 12 respectively. Light sheet width was adjusted to the magnification and thickness was set at its minimum (3.86 μm). Acquisitions were made with 3 light sheets. In some cases, samples were illuminated from both sides. Dynamic focusing was used to ensure constant thickness of the light sheets over large samples. Fluorochromes were excited with 488, 561, or 640 laser. The following filters were used: 525/20, 595/40, and 595/40 to collect the green, red, and far-red emitted light. Image acquisitions were set using the ImspectorPro software (Miltenyi Biotec). Each channel was acquired sequentially. Images were acquired every 2 or 1.5 μm.

### Image visualization using Imaris

The images acquired were processed with Imaris 9.0.2 (Oxford instruments). The channel colors were chosen according to the probe/amplifier pair used. The intensity and contrast were modified by adjusting the min and max gray levels in the display setting function. 3D images of the embryos were generated in the 3D view using the default MIP projection mode. To obtain optical sections of the regions of interest, image stacks were re-oriented using the “free rotate” function and analyzed with the “section view” using the normal mode. The 3D images and 2D sections presented in the figures were taken with the snapshot tool from the 3D view or section view modes.

### Intensity quantification

Unprocessed 16-bit images were quantified in FIJI. Intensity profile was generated with the “plot profile” function. The line used was 20 pixels wide. The intensity profile was exported as an image from FIJI. Mean intensity was measured in regions of interest of same size and placed at different dorsoventral positions. The measures were made on two series of 21 consecutive plans separated by 80 plans from 1 spinal cord. Conversion to Fire LUT was done in FIJI, and calibration bar was added. Graph and statistical tests were performed in PRISM 9. Non parametric ANOVA (Kruskal–Wallis) + post hoc pairwise comparisons (Dunn’s multiple comparisons test) was performed as data distributions were not normal.

### Fluorescence microscopy

Non-cleared embryos were imaged with Axiozoom V16 stereomicroscope (ZEISS) equipped with a × 1 front objective and a × 16 Zoom. The images were captured with the CoolSNAP HQ2 Monochrome camera (Photometrics®) and ZEN software (ZEISS).

### Electroporation

In ovo electroporation of spinal cord neuron was performed on HH14/HH15 chicken embryos as described previously [79]. pCAGEN-GFP was diluted in PBS (DPBS, Gibco, 14190250) to a final concentration of 1.5 μg/μl, and the solution was injected into the lumen of the neural tube using Picopritzer III injector (Micro Control Instrument Ltd., UK). Electrodes (CUY611P7-4, Sonidel) were placed along the back of the embryo, at the thoracic level, and 3 pulses (29 V, 50 ms, 500 ms interpulse) were delivered by CUY-21 generator (Sonidel). Electroporated embryos were then incubated at 38.5 °C.

### Supplementary Information

Additional file 1: Fig. S1 HCR-RNA-FISH gives limited information on gene expression without embryo clearing and protocol optimizations. a, b HCR RNA-FISH images of E3.5 embryo observed under stereomicroscope. HCR RNA-FISHs for *SLIT2*, *ISL1* and *SOX10* in a and *PAX6* and *ASCL1* are shown in b. Arrowheads point to the eye and arrows indicate the spinal cord (SC) region, including the neural crest cells and DRG. c Image of E5.5 ECi cleared embryos fixed without blood and heart removal. Red blood cells retain their pigmentation despite efficient transparization of the tissues. Dashed line outlines the embryo. Arrow head points to the heart. d Comparison of *ISL1* HCR RNA-FISH detection in the spinal cord of E.5.5 whole mount embryos using HH10 (2pmol, entire embryo) or the optimized protocol (4pmol, without head). Note that initial protocol gives low signal-to-noise ratio and fail to detect *ISL1* expression in the Dorsal interneurons (DI) (Arrows). In both cases, the blood and heart were removed. e, f, f’ Images of the HCR RNA-FISH on the E5.5 embryos after one-year storage. e Double HCR RNA-FISH *SLIT2/ISL1* in the spinal cord (SC). *SLIT2* and *ISL1* are both expressed in MNs (arrow heads). *SLIT2* is highly expressed in floor plate (→) and *ISL1* in cranial ganglions ($\to$). f, f’ HCR RNA-FISH for *SLIT2* combined with NF-M immunostaining on the E5.5 limb. Scale bars: 500μm (a, b), 1mm (c), 200μm (d, e), 150μm (f), 100μm (f’).

Additional file 2: Fig. S2 GFP Immunostaining is required to monitor the morphology of GFP-expressing cells. a Endogenous GFP fluorescence is lost during HCR RNA-FISH and ECi Clearing. Images of endogenous GFP signal observed under stereomicroscope before PFA fixation (i), after HCR RNA-FISH (ii) and after ECi clearing (iii). All images were acquired with the same settings. Note the shrinkage of the embryo after ECi clearing (iii). Dashed lines outline the embryo. b, c Virtual coronal sections of a GFP electroporated embryo at E4.5. Dashed line outlines the spinal cord. Images were acquired with the same settings. Laser power is indicated in the gray boxes. Exposure time was 100ms for all channels. b Combination of HCR RNA-FISH without *SLIT2* probes and GFP immunostaining. Detection was made without probes and amplifier (B2-546) was added for the amplification step. Arrow indicates GFP positive cells revealed by immunostaining that cannot be detected in 488 channel. Arrow heads point to autofluorescent cells seen in the 488 channel. c GFP signal after HCR RNA-FISH for *SLIT2* without primary anti-GFP immunostaining. No signal is observed after incubation with secondary antibody. Scale bars: 1mm (a), 100μm (b, c).

Additional file 3: Individual values of the data presented in Figure 3d.

## Data Availability

All data generated or analyzed during this study are included in this published article and its supplementary information files.

## Abbreviations

*ASCL1*: Achaete-scute family BHLH transcription factor 1
BSA: Bovine serum albumin
*CNTN2*: Contactin 2
*CXCR4*: C-X-C motif chemokine receptor 4
DI3: Dorsal interneuron subtype 3
DI: Dorsal interneuron
DM: Dorsal muscle mass
DMSO: Dimethyl sulfoxide
DRG: Dorsal root ganglion
Ex: Embryonic day x
ECi: Ethyl cinnamate
EtOH: Ethanol
FP: Floor plate
GFP: Green fluorescent protein
HHx: Hamburger and Hamilton stage number x
HCR RNA-FISH: RNA fluorescence in situ hybridization
iDISCO: Immunolabeling-enabled three-dimensional imaging of solvent-cleared organs
IF: Immunofluorescent
*ISL1*: ISL LIM homeobox 1
MetOH: Methanol
MN: Motoneuron
*NEFM*: Neurofilament medium
NF-M: Neurofilament medium
ON: Overnight
OV: Otic vesicle
*PAX6*: Paired box 6
PBS: Phosphate-buffered saline
PBST: PBS tween
PFA: Paraformaldehyde
PK: Proteinase K
*ROBO2*: Roundabout guidance receptor 2
RT: Room temperature
SeeDB: See Deep Brain
*SLIT2*: Slit guidance ligand 2
*SOX10*: SRY-box transcription factor 10
SSCT: Saline-sodium citrate with Tween 20X
SC: Spinal cord
