# Supplementary material for: 3D exploration of gene expression in chicken embryos through combined RNA fluorescence in situ hybridization, immunofluorescence, and clearing
Source: BMC Biol. 2024 Jun 3;22:131. doi: 10.1186/s12915-024-01922-0 (PMC11149291; doi:10.1186/s12915-024-01922-0)
Supplement: Supplementary file 1 — Additional file 1: Fig. S1 HCR-RNA-FISH gives limited information on gene expression without embryo clearing and protocol optimizations. a, b HCR RNA-FISH images of E3.5 embryo observed under stereomicroscope. HCR RNA-FISHs for SLIT2, ISL1 and SOX10 in a and PAX6 and ASCL1 are shown in b. Arrowheads point to the eye and arrows indicate the spinal cord (SC) region, including the neural crest cells and DRG. c Image of E5.5 ECi cleared embryos fixed without blood and heart removal. Red blood cells retain their pigmentation despite efficient transparization of the tissues. Dashed line outlines the embryo. Arrow head points to the heart. d Comparison of ISL1 HCR RNA-FISH detection in the spinal cord of E.5.5 whole mount embryos using HH10 (2pmol, entire embryo) or the optimized protocol (4pmol, without head). Note that initial protocol gives low signal-to-noise ratio and fail to detect ISL1 expression in the Dorsal interneurons (DI) (Arrows). In both cases, the blood and heart were removed. e, f, f’ Images of the HCR RNA-FISH on the E5.5 embryos after one-year storage. e Double HCR RNA-FISH SLIT2/ISL1 in the spinal cord (SC). SLIT2 and ISL1 are both expressed in MNs (arrow heads). SLIT2 is highly expressed in floor plate (→) and ISL1 in cranial ganglions ($\to$→). f, f’ HCR RNA-FISH for SLIT2 combined with NF-M immunostaining on the E5.5 limb. Scale bars: 500μm (a, b), 1mm (c), 200μm (d, e), 150μm (f), 100μm (f’). [file 12915_2024_1922_MOESM1_ESM.pdf]

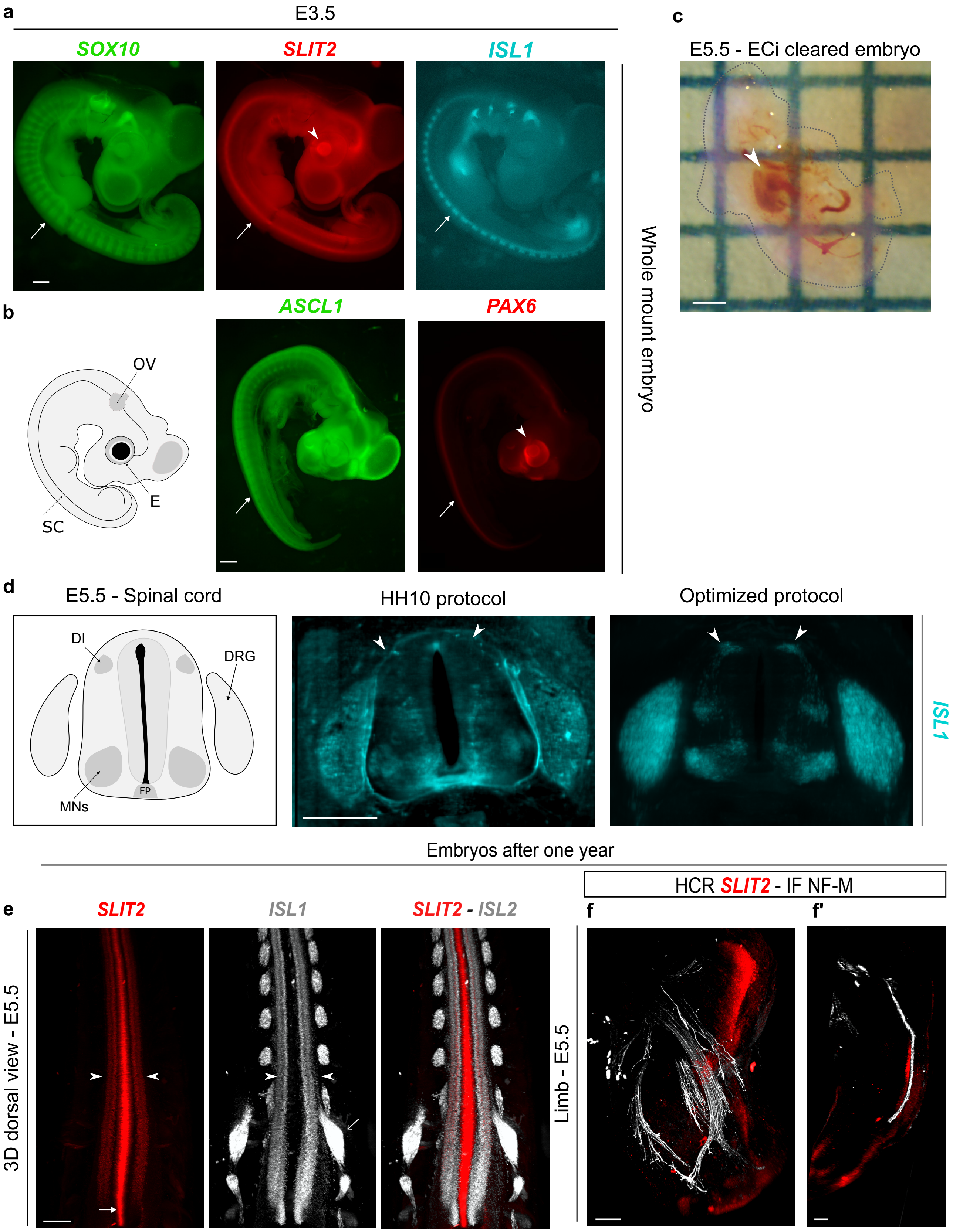

Additional File 1: Fig. S1 HCR-RNA-FISH gives limited information on gene expression without embryo clearing and protocol optimizations.

a, b HCR RNA-FISH images of E3.5 embryo observed under stereomicroscope. HCR RNA-FISHs for *SLIT2*, *ISL1* and *SOX10* in **a** and *PAX6* and *ASCL1* are shown in **b.** Arrowheads point to the eye and arrows indicate the spinal cord (SC) region, including the neural crest cells and DRG. **c** Image of E5.5 ECi-cleared embryos fixed without blood and heart removal. Red blood cells retain their pigmentation despite efficient transparisation of the tissues. Dashed line outlines the embryo. Arrow head points to the heart. **d** Comparison of *ISL1* HCR RNA-FISH detection in the spinal cord of E.5.5 whole mount embryos using HH10 (2pmol, entire embryo) or the optimized protocol (4pmol, without head). Note that initial protocol gives low signal-to-noise ratio and fail to detect *ISL1* expression in the Dorsal interneurons (DI) (Arrows). In both cases, the blood and heart were removed. **e, f, f'** Images of the HCR RNA-FISH on the E5.5 embryos after one-year storage. **e** Double HCR RNA-FISH *SLIT2/ISL1* in the spinal cord (SC). *SLIT2* and *ISL1* are both expressed in MNs (arrow heads). *SLIT2* is highly expressed in floor plate (→) and *ISL1* in cranial ganglions (→). f, f' HCR RNA-FISH for *SLIT2* combined with NF-M immunostaining on the E5.5 limb.

Scale bars: 500μm (a, b), 1mm (c), 200μm (d, e), 150μm (f), 100μm (f')
