# Supplementary material for: 3D exploration of gene expression in chicken embryos through combined RNA fluorescence in situ hybridization, immunofluorescence, and clearing
Source: BMC Biol. 2024 Jun 3;22:131. doi: 10.1186/s12915-024-01922-0 (PMC11149291; doi:10.1186/s12915-024-01922-0)
Supplement: Supplementary file 2 — Additional file 2: Fig. S2 GFP Immunostaining is required to monitor the morphology of GFP-expressing cells. a Endogenous GFP fluorescence is lost during HCR RNA-FISH and ECi Clearing. Images of endogenous GFP signal observed under stereomicroscope before PFA fixation (i), after HCR RNA-FISH (ii) and after ECi clearing (iii). All images were acquired with the same settings. Note the shrinkage of the embryo after ECi clearing (iii). Dashed lines outline the embryo. b, c Virtual coronal sections of a GFP electroporated embryo at E4.5. Dashed line outlines the spinal cord. Images were acquired with the same settings. Laser power is indicated in the gray boxes. Exposure time was 100ms for all channels. b Combination of HCR RNA-FISH without SLIT2 probes and GFP immunostaining. Detection was made without probes and amplifier (B2-546) was added for the amplification step. Arrow indicates GFP positive cells revealed by immunostaining that cannot be detected in 488 channel. Arrow heads point to autofluorescent cells seen in the 488 channel. c GFP signal after HCR RNA-FISH for SLIT2 without primary anti-GFP immunostaining. No signal is observed after incubation with secondary antibody. Scale bars: 1mm (a), 100μm (b, c). [file 12915_2024_1922_MOESM2_ESM.pdf]

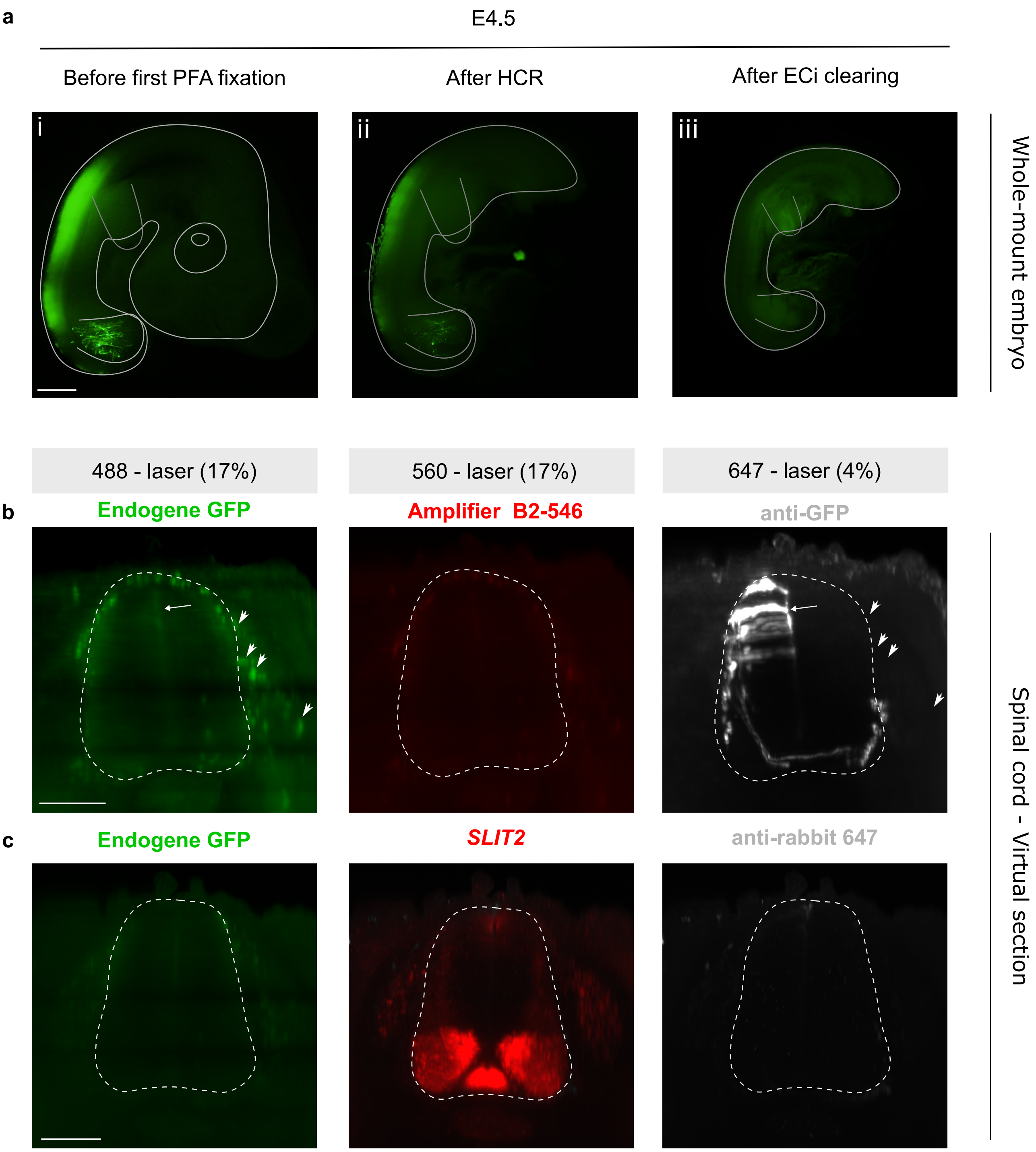

Additional File 2: Fig. S2 GFP Immunostaining is required to monitor the morphology of GFP-expressing cells

a Endogenous GFP fluorescence is lost during HCR RNA-FISH and ECi-Clearing. Images of endogenous GFP signal observed under stereomicroscope before PFA fixation (i), after HCR RNA-FISH (ii) and after ECi clearing (iii). All images were acquired with the same settings. Note the shrinkage of the embryo after ECi-clearing (iii). Dashed lines outline the embryo. **b, c** Virtual coronal sections of a GFP electroporated embryo at E4.5. Dashed line outlines the spinal cord. Images were acquired with the same settings. Laser power is indicated in the gray boxes. Exposure time was 100ms for all channels. **b** Combination of HCR RNA-FISH without *SLIT2* probes and GFP immunostaining. Detection was made without probes and amplifier (B2-546) was added for the amplification step. Arrow indicates GFP positive cells revealed by immunostaining that cannot be detected in 488 channel. Arrow heads point to autofluorescent cells seen in the 488 channel. **c** GFP signal after HCR RNA-FISH for *SLIT2* without primary anti-GFP immunostaining. No signal is observed after incubation with secondary antibody. Scale bars: 1mm (a), 100µm (b, c)
